# Effect of *CYP2B6* Gene Polymorphisms on Efavirenz Plasma Concentrations in Chinese Patients with HIV Infection

**DOI:** 10.1371/journal.pone.0130583

**Published:** 2015-06-24

**Authors:** Xianmin Meng, Kang Yin, Jiangrong Wang, Ping Dong, Li Liu, Yinzhong Shen, Li Shen, Qing Ma, Hongzhou Lu, Weimin Cai

**Affiliations:** 1 Department of Clinical Pharmacy and Pharmaceutical Administration, School of Pharmacy, Fudan University, Shanghai, China; 2 Department of Pharmacy, Shanghai Public Health Clinical Center, Shanghai, China; 3 Department of Infectious Disease, Shanghai Public Health Clinical Center, Shanghai, China; 4 Department of Pharmacy Practice, School of Pharmacy and Pharmaceutical Sciences, University at Buffalo, Buffalo, New York, United States of America; University of Texas Health Science Center San Antonio Texas, UNITED STATES

## Abstract

**Objectives:**

The main aim of this study was to investigate the effect of *CYP2B6* gene polymorphisms on efavirenz (EFV) plasma concentrations in Han Chinese patients with human immunodeficiency virus (HIV) infection.

**Methods:**

In total, 322 patients were recruited for study. EFV plasma concentrations at steady-state were determined using high-performance liquid chromatography. Genotyping for seven single nucleotide polymorphisms (SNPs), including 171+967C>A, 171+3212C>T, 171+4335T>C, 516G>T, 785A>G, 1295-913G>A, and *1355A>G of *CYP2B6*, was performed using ligase detection reaction (LDR). SPSS 18.0 and Haploview 4.2 were applied for statistical analyses.

**Results:**

The average EFV concentration of patients was 2.35±2.09 μg/mL. Overall, 22% patients displayed EFV concentrations out of the therapeutic range of 1–4 μg/mL (13.1% < 1 μg/mL, 9.3% > 4 μg/mL). We observed significant association of 171+967C>A, 171+4335T>C, 516G>T, 785A>G and *1355A>G with high plasma EFV levels (*p*<.01). The predictive accuracy values of 171+4335CC, 516TT and 785GG for EFV concentrations > 4 μg/mL were 56.7%, 56.7% and 60%, respectively. We observed strong linkage disequilibrium for 171+967C>A, 171+4335T>C, 516G>T and 785A>G, resulting in five haplotypes. The frequencies of the five haplotypes (high to low) were as follows: CCTG (0.328), ACTG (0.280), ACCT (0.189), ATTG (0.186) and ACCG (0.017). The frequency of CCTG (0.524) in patients with EFV plasma concentrations < 1 μg/mL was significantly higher than that in other patient groups, while that of ACCT (0.733) was significantly higher in patients with EFV concentrations > 4 μg/mL, relative to other patient groups. Average EFV concentrations of patients carrying ACTG (1.78 μg/mL), ACCT (7.50 μg/mL), and ATTG (1.92 μg/mL) haplotypes were markedly higher than those of patients carrying the CCTG haplotype. The predictive accuracy of ACCT for EFV > 4 μg/mL was 81%.

**Conclusions:**

Chinese patients administered standard doses of EFV require therapeutic drug monitoring or personalized medication management. Based on the current findings, we propose that 171+4335T>C, 516G>T, 785A>G and haplotype ACCT may be effectively used as genomic markers for EFV, which should aid in improving the efficacy of EFV-containing treatments and reduce the incidence of adverse reactions.

## Introduction

Efavirenz (EFV) is an important first-line drug for HIV-infected patients in China and widely used globally, particularly in developing countries, owing to its excellent efficacy [1]. Several studies have demonstrated considerable intra- and inter-patient variations in plasma concentrations and a narrow therapeutic window (1–4 μg/mL) of EFV. Moreover, the EFV plasma concentration is closely related to antiviral effects and adverse reactions [2–5]. Patients may be more likely to experience treatment failure or viral resistance at plasma concentrations < 1 μg/mL. Conversely, increased adverse reaction incidence, such as central nervous system toxicity, has been reported at EFV concentrations > 4 μg/mL [4, 6, 7].

EFV is mainly metabolized by the CYP P-450 isoenzyme, CYP2B6, in liver [8]. The *CYP2B6* gene located on chromosome 19 is highly polymorphic, with significantly different allele frequencies among various ethnic populations. Taking *CYP2B6* 516G>T (rs3745274) as an example, allele frequency varies from 0.18 to 0.35 in the Chinese population, and is maintained at a relatively high level [9–12]. Mutations of the *CYP2B6* gene affect the expression and enzyme activity of the translated protein, resulting in significant differences in the pharmacokinetics of EFV among individuals and races, in turn, leading to variations in efficacy and toxicity [2, 13, 14]. In recent years, researchers have investigated several SNPs of *CYP2B6*, including 516G>T, 785A>G (rs2279343), 983T>C (rs28399499), and 1459C>T (rs3211371), which are all known to be associated with EFV plasma concentrations [2, 15–21]. However, single SNP analysis may not provide sufficient accuracy to predict individual differences of EFV plasma concentrations. Considerable research attention has focused on attempting to integrate the effects of several SNPs that reduce the metabolic function of CYP2B6, with a view to increasing prediction accuracy [22, 23].

However, limited studies to date have examined the correlation between EFV plasma concentrations and *CYP2B6* gene polymorphisms in Han Chinese HIV-infected patients [11, 24]. An earlier report showed that the percentage of Han Chinese HIV-infected patients with EFV concentrations > 4 μg/mL was 28.6% after oral administration of EFV 600 mg once daily for at least two weeks [25]. The EFV concentrations of patients with 516 GT and TT genotypes were significantly higher than those of GG genotype patients [11, 24]. In clinical practice, EFV treatment has been withdrawn in many Han Chinese HIV-infected patients because of severe toxic reactions, which impact the efficacy of antiretroviral therapy and cause negatively affect to subsequent treatments. In the current study, we investigated the effects of *CYP2B6* gene polymorphisms on EFV plasma concentrations in Han Chinese patients with HIV infection, with the aim of providing valuable data supporting the requirement for individualized medication.

## Materials and Methods

### Patients

In total, 322 Han Chinese HIV-infected outpatients receiving EFV combination antiretroviral therapy (cART) at Shanghai Public Health Clinical Center from January 2012 to January 2013 were enrolled in this study. All subjects, including 291 males and 31 females, were adults with an average age of 40 years (range: 18 to 78 years). Mean height and weight were recorded as 171 ± 6 cm and 63±9 kg (BMI 21.5 ± 2.6), respectively.

Subjects received EFV (600 mg once daily)-containing cART for at least 2 weeks and were advised not to take other medications that could reduce or induce isozymes of cytochrome P450, such as rifampicin. The average duration of cART was 17 months. The cART regimens included: (1) zidovudine (AZT, 300 mg twice daily), lamivudine (3TC, 300 mg daily) and EFV, (2) stavudine (d4T, 30 mg twice daily), 3TC and EFV, (3) tenofovir (TDF, 300 mg daily), 3TC and EFV, (4) TDF, lopinavir/ritonavir (LPV/r, 400/100 mg twice daily) and EFV, (5) d4T, TDF and EFV, (6) 3TC, LPV/r and EFV, (7) AZT, EFV and LPV/r. This study followed the principles of the Declaration of Helsinki, and approval was granted by the Ethics Committee of Shanghai Public Health Clinical Center. Written informed consent was obtained from all subjects.

Whole blood samples (5 mL) at 12–16 h post-dose were collected using EDTA anticoagulant tubes for determining the concentration of EFV and *CYP2B6* genotyping [26]. Plasma samples were heat-deactivated in a 56°C water bath for 60 min and stored at −80°C before analysis. Demographic and related data were collected, including age, weight, height, gender, cART regimens, dose and time of EFV administration, and sampling time.

### Quantification of EFV concentration

EFV plasma concentrations were determined using reverse-phase high-performance liquid chromatography (RP-HPLC) with ultraviolet (UV) detection based on a previously described protocol, with minor modifications [27]. HPLC was performed using Shimadzu LC-20A consisting of a column compartment CTO-20A, degasser DGU-20A5, pump CBM-20A, auto-sampler SIL-20AC, SPD-20AV UV detector, and YMC-Pack ODS-A column (C18, 150 mm × 4.6 mm, 5 μm) with a guard column (ZORBAX Eclipse Plus-C18). The mobile phase comprised 62% acetonitrile, 38% 0.01 mol/L NaH2PO4 buffer (containing 0.01 mol/L triethylamine, pH 5.2). Plasma proteins were precipitated with acetonitrile (containing 1.0 μg/mL diazepam as the internal standard) before centrifugation at 15000 rpm for 6 min, and the supernatant injected directly into the machine. The UV detector was set at 247 nm and the injection volume as 20 μL. The chromatogram was run for 7.5 min at a flow rate of 1.0 mL/min at 30°C. EFV and the internal standard (diazepam) were separated. Retention times for diazepam and EFV were 4.535 and 6.475 min, respectively. The linear range was 0.10–20 μg/mL, with intraday/interday coefficient of variation of 1.9/7.2%, 2.4/2.2% and 2.6/2.2% at concentrations of 0.3, 3.0, 10.0 μg/mL, respectively. The lower limit of quantitation was 89 ng/mL.

### SNP selection and genotyping

SNPs were selected primarily based on: 1) data on the *CYP2B6* gene among the Han Chinese population obtained from http://www.ncbi.nlm.nih.gov/SNP and filtered using Haploview 4.2., and 2) SNPs of *CYP2B6* that had a significant influence on EFV plasma concentrations in previous reports. In total, 7 SNPs of *CYP2B6* were selected for study, including 171+967C>A (rs2099361), 171+3212C>T (rs4803415), 171+4335T>C (rs1872125), 516G>T, 785A>G, 1295-913G>A (rs7260329), and *1355A>G (rs707265). All genotyping experiments were performed by Shanghai BioWing Applied Biotechnology (www.biowing.com.cn). Genomic DNA was isolated from peripheral blood using an AxyPrep-96 (AXYGEN) kit, and target DNA sequences amplified using a multiplex polymerase chain reaction (PCR) method. After PCR, genotyping was carried out using an oligonucleotide ligation detection reaction (LDR)-fluorescent microsphere assay. LDR conditions were as follows: 95°C for 2 min, 94°C for 30 s, and 50°C for 2 min (35 cycles). Fluorescent LDR products were differentiated using ABI Sequencer 377. The primers used are summarized in Table 1. Templates containing two alleles in each SNP were synthesized as positive controls. PCR-LDR findings were confirmed by sequencing the PCR products of 30 samples for each SNP.

**Table 1:** Primer sequences and lengths for PCR amplicons of SNPs.

Rs ID	Polymorphisms	Location	Forward Primer (5’-3’)	Reverse Primer (5’-3’)	Product Size (bp)
rs2099361	c.171+967C>A	intron 1	ACCTGTAGTTCCAGCTACTT	CACCATCATAATGGACTTGTC	302
rs4803415	c.171+3212C>T	intron 1	TTTACCCATAAGTCTGCAT	AATAATGGCTGCAAAAGGCATATTT	364
rs1872125	c.171+4335T>C	intron 1	GCCATCAATCAATAATACCTGA	TGTATGTCTGGCTGAACCGGTGA	337
rs3745274	c.516G>T	exon 4	GTCAAATTACTCAGCCTCTCG	GTCTGGTAGAACAAGTTCAGCA	382
rs2279343	c.785A>G	exon 5	AGGCAAGTTTACAAAAACCTG	CCCTCCCTAGTCTTTCTTCTTCC	255
rs7260329	c.1295-913G>A	intron 8	CCTTCTGGGTATGCCAAAGGGATG	CTAAGGAGGCTTAAGGTTTGGTTAC	222
rs707265	c.*1355A>G	exon 9	TATGTGATCTTTTGTGTCTGGTTG	GCATTGAGGTGAGAGAGGCA	374

### Statistical analysis

Continuous data, such as age, height, BMI, course of treatment of antiviral therapy and EFV concentration, were summarized as mean values ± SD or mean and 95% confidence interval (95% CI). Statistical analysis was performed using SPSS 18.0. Normality of EFV concentration data was assessed with the Kolmogorov–Smirnov method. Student’s t-test and single factor analysis of variance, Mann–Whitney and Kruskal–Wallis tests were employed for comparison. SNP frequencies were calculated using the observed numbers of alleles for each SNP. Genotypes were tested for Hardy–Weinberg equilibrium with the chi-square test of observed versus predicted genotype frequencies (from allele frequency). Haplotype structures and their frequencies were estimated from the observed number of genotypes using Haploview 4.2 [28]. Haplotype frequencies were examined with the Chi-square test. Analyses were two-sided, and the results considered significant at *p*-values below 0.05.

## Results

### Demographics, cART regimens and association with EFV plasma concentrations

The average EFV concentration of the 322 subjects was 2.35 ± 2.09 μg/mL (95% CI: 2.12–2.58 μg/mL), among which 78% (n = 250), 13% (n = 42) and 9% (n = 30) were within, below and above the proposed therapeutic window of 1–4 μg/mL, respectively. Demographics and cART regimens are summarized in Table 2. Univariate analysis revealed no significant effects of gender, age, BMI or cART regimens on EFV concentrations (*p*>0.05).

**Table 2:** Demographic data and cART regimens and effects on EFV plasma concentrations.

Factors	Categories	N (%)	Mean ± SD (μg/mL)	95%CI (μg/mL)	*p*
Gender	Male	291 (90)	2.30±2.01	2.07–2.53	0.357
	Female	31 (10)	2.79±2.70	1.80–3.78	
Age	<60	298 (92)	2.36±2.08	2.12–2.59	0.374
	≥60	24 (8)	2.27±2.15	1.37–3.18	
BMI	BMI <25	288 (89)	2.33±1.99	2.11–2.57	0.266
	BMI ≥25	34 (11)	2.45±2.80	1.47–3.43	
Regimen	AZT+3TC+EFV	186 (58)	2.36±2.14	2.05–2.67	0.143
	d4T+3TC+EFV	82 (25)	2.45±2.30	1.94–2.96	
	TDF+3TC+EFV	48 (15)	2.07±1.53	1.63–2.52	
	Others	6 (2)	2.90±1.09	1.75–4.05	

BMI, body mass index; cART, combination antiretroviral therapy; AZT, zidovudine; 3TC, lamivudine; EFV, efavirenz; d4T,stavudine; TDF, tenofovir.

### Allele and genotype frequencies of *CYP2B6* gene SNPs and association with EFV plasma concentrations

**Fig 1:**
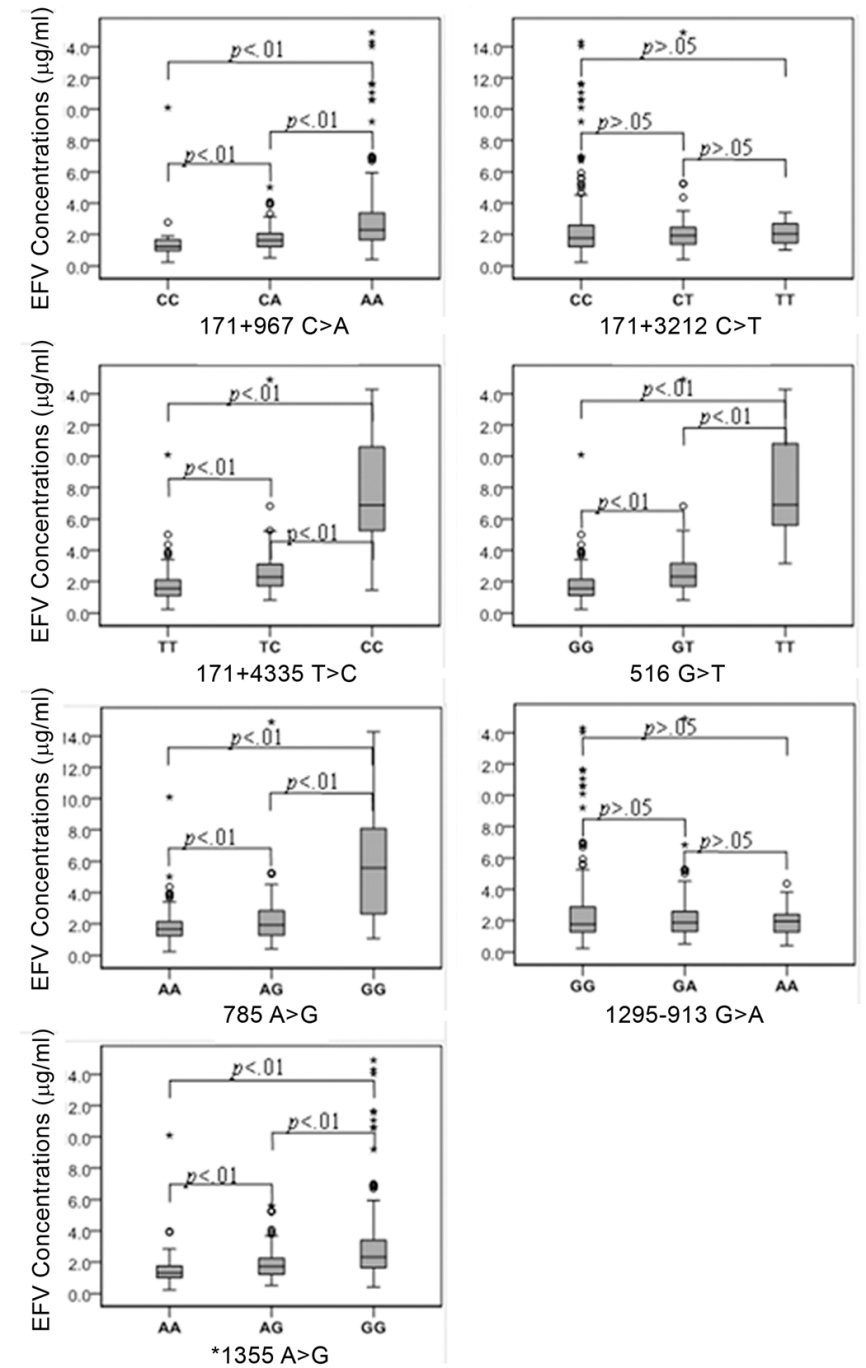
Effects of 7 *CYP2B6* SNPs on EFV plasma concentrations of HAN Chinese HIV-infected patients. Values are presented as mean±SD. 171+967C>A, 171+4335T>C, 516G>T, 785A>G and *1355A>G were significantly associated with high EFV plasma concentrations (*p* < .01), but not 171+3212C>T and 1295-913G>A (*p*>.05).

All 7 SNPs of *CYP2B6* conformed to Hardy–Weinberg equilibrium and were further analyzed with Haploview 4.2. The allele and genotype frequencies of SNPs and association with EFV concentrations are summarized in Table 3. Univariate analysis showed that 171+967C>A, 171+4335T>C, 516 G>T, 785A>G and *1355A>G are significantly associated with high plasma EFV levels (*p*<0.01), but not 171+3212C>T or 1295-913G>A (*p*>.05) (Table 3; Fig 1). The genotype distribution of 30 patients with EFV concentrations > 4 μg/mL is summarized in Table 4. In the patient group with EFV concentrations > 4 μg/mL, 89.5% (17/19) displayed the 516 TT genotype, followed by 171+4335TT (81.0%, 17/21), 785AA (58.1%, 18/31), *1355AA (19.2%, 23/120) and 171+967CC (18.1%, 27/149). Genotype frequencies of 516TT, 171+4335TT and 785AA in 30 subjects with EFV concentrations > 4 μg/mL were 56.7%, 56.7% and 60.0%, respectively.

**Table 3:** Allele and genotype frequencies of *CYP2B6* SNPs and their relationship with EFV plasma concentrations in 322 Chinese patients with HIV infection.

SNPs	Alleles	Allele Frequencies		Genotype Frequencies–n (%)			Mean EFV Concentration (95%CI) -μg/mL			*p*
	(A1/A2)	A1	A2	A1/A1	A1/A2	A2/A2	A1/A1	A1/A2	A2/A2	
c.171+967C>A	C/A	0.328	0.672	38(11.8)	135(41.9)	149(46.3)	1.48(0.99–1.99)	1.71(1.58–1.84)	3.15(2.71–3.58)	<0.01
c.171+3212C>T	C/T	0.814	0.186	215(66.8)	94(29.2)	13(4.0)	2.47(2.16–2.78)	2.12(1.79–2.46)	2.02(1.57–2.48)	0.932
c.171+4335T>C	T/C	0.793	0.207	210(65.2)	91(28.3)	21(6.5)	1.71(1.58–1.84)	2.63(2.28–2.99)	7.50(5.78–9.22)	<0.01
c.516G>T	G/T	0.811	0.189	219(68.0)	84(26.1)	19(5.9)	1.72(1.59–1.85)	2.68(2.30–3.06)	8.12(6.49–9.76)	<0.01
c.785A>G	A/G	0.736	0.264	183(56.8)	108(33.6)	31(9.6)	1.79(1.65–1.94)	2.26(1.95–2.58)	5.95(4.50–7.40)	<0.01
c.1295-913G>A	G/A	0.545	0.455	97(30.1)	157(48.8)	68(21.1)	3.03(2.40–3.66)	2.12(1.89–2.36)	1.90(1.70–2.10)	0.792
c.*1355A>G	A/G	0.390	0.610	49(15.2)	153(47.5)	120(37.3)	1.63(1.21–2.05)	1.86(1.70–2.01)	3.27(2.75–3.79)	<0.01

Abbreviations: 95% CI, 95% confidence interval.

**Table 4:** Genotypes and frequencies of *CYP2B6* SNPs in patients with EFV plasma concentrations >4 μg/mL (n = 30).

A. Individual Data		Genotype							
Patient #	EFV (μg/mL)	171+967C>A		171+4335T>C		516G>T		785A>G	*1355A>G
1	4.087	C/A		C/T		G/T		A/G	A/G
2	4.0971	A/A		C/T		G/T		A/G	G/G
3	4.2348	A/A		C/T		G/T		A/G	G/G
4	4.3652	A/A		T/T		G/G		A/A	G/G
5	4.453	A/A		C/T		G/T		A/G	G/G
6	4.5176	A/A		C/T		G/T		A/G	G/G
7	4.6536	A/A		C/C		T/T		G/G	G/G
8	5.0084	C/A		T/T		G/G		A/A	G/G
9	5.2007	A/A		C/T		G/T		A/G	G/G
10	5.2288	A/A		C/T		G/T		A/G	A/G
11	5.253	A/A		C/C		T/T		G/G	A/G
12	5.2677	A/A		C/T		G/T		A/G	A/G
13	5.5801	A/A		C/C		T/T		G/G	A/G
14	5.5978	A/A		C/C		T/T		G/G	A/G
15	5.9313	A/A		C/C		T/T		G/G	G/G
16	6.6994	A/A		C/C		T/T		G/G	G/G
17	6.8276	A/A		C/T		G/T		G/G	G/G
18	6.879	A/A		C/C		T/T		G/G	G/G
19	6.9154	A/A		C/C		T/T		G/G	G/G
20	6.9984	A/A		C/C		T/T		G/G	G/G
21	9.199	A/A		C/C		T/T		G/G	G/G
22	10.096	C/C		T/T		G/G		A/A	A/A
23	10.5778	A/A		C/C		T/T		G/G	G/G
24	10.596	A/A		C/C		T/T		G/G	G/G
25	11.0563	A/A		C/C		T/T		G/G	G/G
26	11.5756	A/A		C/C		T/T		G/G	G/G
27	11.6234	A/A		C/C		T/T		G/G	G/G
28	14.03	A/A		C/C		T/T		G/G	G/G
29	14.285	A/A		C/C		T/T		G/G	G/G
30	14.892	A/A		C/T		G/T		A/G	G/G
**B. Summary**									
**SNPs**	**Alleles (A1/A2)**	**A1/A1 (n)**		**A1/A2 (n)**		**A2/A2 (n)**		**Ratio α (%)**	**Ratio β (%)**
		**Total**	**High**	**Total**	**High**	**Total**	**High**		
171+967C>A	C/A	38	1	135	2	149	27	18.1	90.0
171+4335T>C	T/C	210	3	91	10	21	17	81.0	56.7
516G>T	G/T	219	3	84	10	19	17	89.5	56.7
785A>G	A/G	183	3	108	9	31	18	58.1	60.0
*1355A>G	A/G	49	1	153	6	120	23	19.2	76.7

**Notes:** High **>**4 μg/mL, Ratio α = >4 μg/mL (A2/A2)/total(A2/A2)/×100%, Ratio β = >4 μg/mL (A2/A2)/30×100%

### *CYP2B6* haplotype frequency and association with EFV concentrations

**Fig 2:**
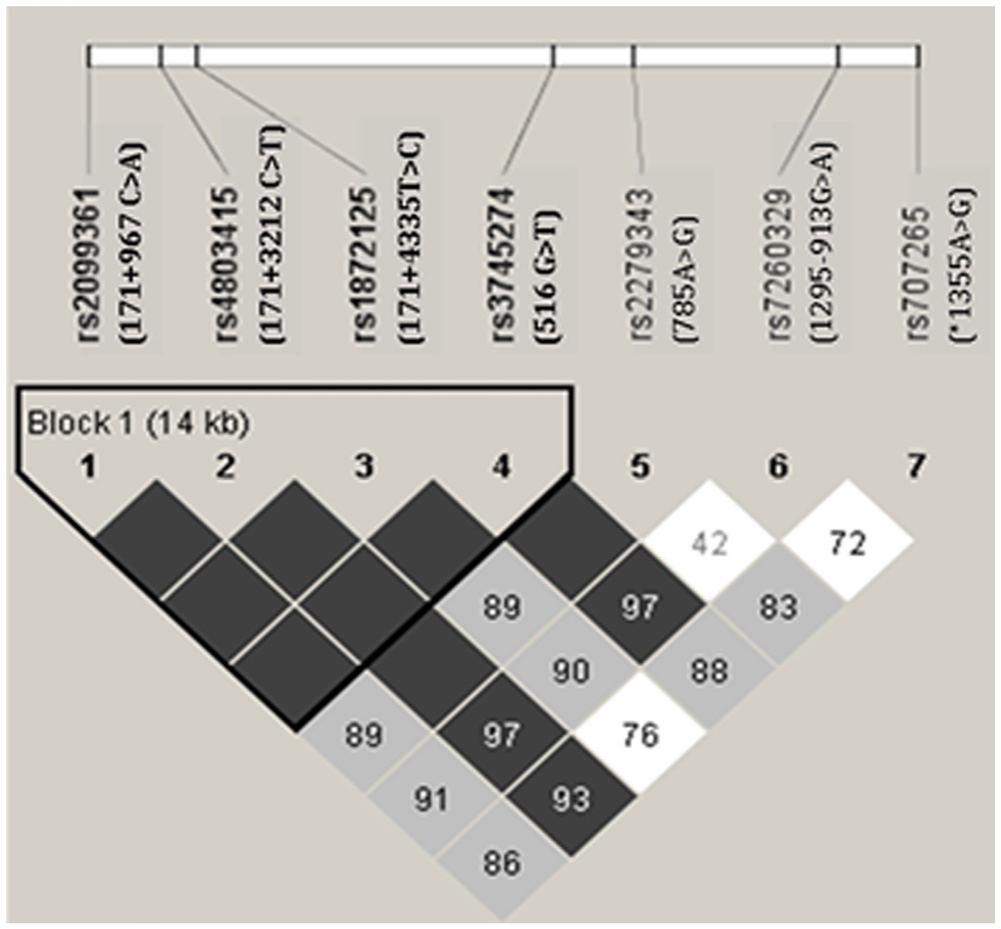
LD Analysis of 7 SNPs of *CYP2B6*. Dark grey squares: strong evidence of LD, light grey squares: uninformative, white squares: strong evidence of recombination. We observed strong linkage disequilibrium among the four SNPs (171+967 C>A, 171+3212 C>T, 171+4335 T>C, and 516 G>T).

Linkage disequilibrium analysis revealed that the D′ value between any two SNPs of 171+967C>A, 171+3212C>T, 171+4335T>C, and 516G>T is 1. The r2 values between 171+967C>A and 171+3212C>T, 171+967C>A and 171+4335T>C, and 171+967C>A and 516G>T were 0.112, 0.127, 0.114, respectively. The r2 values between 171+3212C>T and 171+4335T>C, and 171+3212C>T and 516G>T were 0.06 and 0.054, respectively, and that between 171+4335T>C and 516G>T was 0.898. These findings indicate strong linkage disequilibrium between the four SNPs (Fig 2). Five haplotypes were made up by the four SNPs. The haplotype with the highest frequency was CCTG (0.328), followed by ACTG (0.280), ACCT (0.189), ATTG (0.186), and ACCG (0.017). The cumulative frequency of the five haplotypes was 100%. The frequency of the CCTG haplotype (0.524) in patients with EFV plasma concentration < 1 μg/mL was significantly higher than that that in other patients, while the frequency of the ACCT haplotype (0.733) in patients with EFV concentrations > 4 μg/mL was significantly higher (Table 5). This finding indicates that patients carrying the CCTG haplotype have a greater likelihood of displaying EFV concentrations < 1 μg/mL while those carrying the ACCT haplotype have a high probability of exhibiting EFV concentrations > 4 μg/mL.

**Table 5:** Haplotypes frequencies in 322 Chinese HIV-infected patients with different EFV plasma concentrations.

Haplotypes	Expected Frequencies (n)	Frequencies (n)		
		<1 μg/mL (n = 42)	1–4 μg/mL (n = 250)	>4 μg/mL (n = 30)
CCTG	0.328 (106)*	0.524(22)	0.332(83)*	0.067(2)**
ACTG	0.280 (90)	0.262(11)	0.290(73)	0.133(4)
ACCT	0.189 (61)**	0.047(2)**	0.150(38)**	0.733(22)
ATTG	0.186 (60)	0.143(6)	0.208(52)	0.067(2)
ACCG	0.017 (5)	0.024(1)	0.020(5)	0.000(0)

Notes: (1) Haplotypes were constructed based on 4 SNPs (171+967C>A, 171+3212C>T, 171+4335T>C and 516G>T).

(2) * *p* < .01, ** *p*< .05.

Data in table 6 showed that ACTG (40.4%) was the most common haplotype in patients, followed by CCTG (32.0%), ATTG (14.9), ACCT (6.5%) and ACCG (2.2%). The average EFV concentrations of patients carrying ACTG (1.78 μg/mL), ACCT (7.50 μg/mL) and ATTG (1.92 μg/mL) haplotypes were significantly higher than those of patients carrying the CCTG haplotype (1.57 μg/mL). The predictive accuracy of ACCT for EFV concentrations > 4 μg/mL was 81%.

**Table 6:** Relationship between haplotypes and EFV plasma concentrations in 322 Chinese patients with HIV infection.

Haplotype	Cases-n (%)	Patients with different EFV concentration–n (%)			Mean EFV concentration –μg/mL (95%CI)	*p*
		<1 μg/mL	1–4 μg/mL	>4 μg/mL		
CCTG	103(32.0)	22(21.4)	79(76.7)	2(1.9)	1.57(1.36–1.79)	—
ACTG	130(40.4)	22(16.9)	106(81.5)	2(1.5)	1.78(1.64–1.93)	= .004
ACCT	21(6.5)	0(0.0)	4(19.0)	17(81.0)	7.50(5.78–9.22)	= 5.425×10^-11^
ATTG	48(14.9)	4(8.3)	43(89.6)	1(2.1)	1.92(1.70–2.15)	= 3.280×10^-4^
ACCG	7(2.2)	1(14.3)	6(85.7)	0(0.0)	1.65(1.19–2.11)	= .279

Notes: (1) Haplotypes were constructed based on 4 SNPs (171+967C>A, 171+3212C>T, 171+4335T>C and 516G>T). (2) Comparing with haplotype “CCTG”. (3) Comparing among the 5 haplotypes, *p* = 2.87×10^-12^.

## Discussion

Numerous studies have focused on the relationship between *CYP2B6* gene polymorphisms and EFV pharmacokinetics in HIV-infected patients of different ethnicities [29–32]. However, limited reports are available on the relationship between *CYP2B6* SNPs and EFV concentrations in the Han Chinese HIV-infected population. The sample sizes of the two earlier published studies were relatively small [11, 25]. To our knowledge, the current study has provided the first comprehensive genetic analysis of *CYP2B6* polymorphisms and their association with EFV concentrations in the largest Chinese HIV-infected patient cohort (n = 322). The average EFV concentration in this study cohort was 2.35 μg/mL, consistent with results from two other studies on HIV-infected Chinese patients [11, 24], which were slightly higher than that reported from a study on Spanish patients (2.27 μg/mL) [33]. Despite the finding that EFV concentrations of the majority of patients (78%) were within the therapeutic window, 72 (22%) had suboptimal EFV concentrations, with 13% < 1 μg/mL and 9% > 4 μg/mL, implying a significant challenge of managing EFV-based regimens in HIV-infected Chinese patients, since EFV concentrations are associated with risk of treatment failure and toxicity, particularly neurotoxicity. It is unlikely that demographic factors, including age, gender and BMI, contribute substantially to the high inter-patient variability in EFV (Table 2). In terms of cART regimens, the most commonly used was AZT+3TC+EFV (58%), followed by d4T+3TC+EFV (25%) and TDF+3TC+EFV (15%). Though these regimens are common in China and other developing countries, no significant drug interactions with EFV of cART regimens used in this study have been reported, suggesting a limited impact on EFV metabolism and concentrations.

Genotype distributions and allele frequencies were in Hardy–Weinberg equilibrium for all 7 *CYP2B6* SNPs analyzed. The observed allele frequencies of 171+967C>A, 171+3212C>T, 171+4335T>C, 516G>T, 785A>G, 1295-913G>A, and *1355A>G were 0.672, 0.186, 0.207, 0.189, 0.264, 0.455, and 0.610, respectively, consistent with earlier reports on Chinese populations [9–12]. In particular, the allele frequencies of 516G>T (0.189) and 785A>G (0.264) were similar to those recorded for other Asian populations, such as Japanese and Korean [23, 34], but lower than that in African populations [17, 18]. Five SNPs, including 171+967C>A (intron 1), 171+4335T>C (intron 1), 516G>T (exon 4), 785A>G (exon 5), and 1295-913G>A (exon 9), were significantly associated with EFV concentrations, suggesting that variations in splicing, coding, and subsequent CYP2B6 enzymatic activity affect EFV metabolism. Notably, variants with decreased enzymatic activity lead to elevated EFV concentrations, as observed in the present study. Associations between 516G>T and EFV pharmacokinetics have been documented in a number of recent studies. Our findings on allele frequency and different genotypes, 68% (GG), 26% (GT) and 6% (TT), were similar to previous observations. Among 19 patients with 516TT, 89.5% (n = 17) had EFV concentrations above 4 μg/mL, with an average of 8.12 μg/ml, ~5- and 3-fold higher than those with 516GG (1.68 μg/mL) and 516GT (2.56 μg/mL), respectively, suggesting a significant impact of 516G>T to EFV metabolism among the Chinese population, consistent with previous findings in Asian and other populations [10, 12, 32, 33]. In addition, 171+4335T>C and 785A>G were significantly associated with mean EFV concentration, which was 2–3 and 3–4 times higher in patients with the homozygous variant genotype than those in the heterozygous and wild-type groups, respectively. The percentages of patients homozygous for 171+4335T>C and 785A>G with EFV concentrations > 4 μg/mL were 81% and 58%, respectively. To our knowledge, this is the first report of an association of 171+4335T>C with EFV concentrations within the Chinese population, which appears as important as 516 G>T. Since the 171+4335T>C polymorphism is common among Chinese and Asian populations, its impact on EFV concentrations warrants further investigation. The high probability of EFV concentrations above 4 μg/mL in relation to homozygous 171+4335T>C (81.0%), 516G>T (89.5%) or 785A>G (58.1%) (Table 4), and high respective predictive accuracy of 56.7%, 56.7% and 60% support the potential utility of these markers to guide dose selection and adjustment of EFV therapy. Although 90% subjects (27/30) with homozygote (A/A) at 171+967C>A had EFV concentrations greater than 4 μg/ml, a ratio higher than 57% (17/30) with 516T/T, they only accounted for 18% among a total of 149 subjects with 171+967A/A whereas the 17 subjects with 516T/T accounted for 90% of the total (n = 19). Thus, the overall prediction potential of 516G>T, 51% (90%*57%), was remarkably higher than that of 171+967C>A, 16% (18%*90%), suggesting a more important role of 516G>T in predicting EFV concentrations.

Haplotype analysis collectively evaluates the interactions of multiple SNPs, leading to a decrease in the metabolic function of CYP2B6. In theory, haplotype accuracy may be higher, compared with single SNPs in predicting EFV pharmacokinetics [22]. To our knowledge, this study represents the first report of relationship between haplotype and EFV concentrations in Chinese patients. Linkage disequilibrium among 171+967C>A, 171+3212C>T, 171+4335T>C and 516G>T was observed, resulting in five haplotypes among which CCTG had the highest frequency (0.328), followed by ACTG (0.280), ACCT (0.189), ATTG (0.186) and ACCG (0.017). The CCTG haplotype was associated with low EFV concentrations (<1 μg/mL), with a calculated a frequency of 0.524 that is significantly higher than other haplotypes (*p* < .05), suggesting predictive value of EFV concentrations <1 μg/mL. Conversely, the high ACCT haplotype frequency (0.733) among patients with EFV concentrations > 4 μg/mL suggests an association with high EFV concentrations.

DNA of patients is composed of diplotypes. It was difficult for us to measure the diplotype for each patient accurately, due to lack of proper equipment. We are able to determine diplotypes of patients, such as CCTG/CCTG, CCTG/ACTG, if the 4 SNPs (171+967 C>A, 171+3212 C>T, 171+4335 T>C, 516 G>T) are all homozygous or only one in four is a heterozygote. However, it is impossible to determine the type of diplotype for patients with two or more heterozygotes of the above SNPs. Based on genotype data of the four SNPs, we calculated that the number of patients with CCTG, ACTG, ACCT, ATTG, and ACCG haplotypes are 103 (32.0%), 130 (40.4%), 21 (6.5%), 48 (14.9%) and 7 (2.2%), respectively. The average EFV concentration of patients carrying ACCT (7.50 μg/mL) was significantly higher than those of patients carrying the CCTG haplotype (1.57 μg/mL). Moreover, the predictive accuracy of ACCT (81%) was relatively higher than that of single SNPs, such as 171+4335T>C (57%), 516G>T (57%) and 785A>G (60%).

Our current study has potential limiting and confounding factors commonly associated with cross-sectional study design. Despite the relatively large sample sizes and multiple SNPs, drug concentration sampling and the nature of combination antiretroviral therapy may interfere with data interpretation. However, the effects of non-compliance and inter-individual variability may be mitigated by selection of patients stabilized with EFV for at least 2 weeks. In addition, the sample collection window was set between 12 and 16 h after EFV administration, which is a relatively large time range [26]. A large proportion of Chinese patients with HIV infection have co-infections with hepatitis B or C virus and tuberculosis (TB). Combinations of drugs may significantly affect the EFV concentration [35]. Indeed, some of the participants (~10%) in this study were co-infected with HIV/TB. The impact of co-infection and concurrent medications for TB, *i*.*e*., rifampin, warrants further investigation.

In summary, a large proportion of the Chinese patients in our study (~22%) displayed EFV concentrations out of the therapeutic window, suggesting potentially high risk of treatment failure or toxicity. Significant associations between SNPs in *CYP2B6* and EFV concentrations were evident, particularly for 171+4335T>C, 516G>T, and 785A>G. Haplotype analysis suggested strong association of CCTG or ACCT and EFV concentrations. Therefore, personalization of medical care may become feasible if these genomic markers are validated and incorporated in EFV-containing treatments in the future. This would improve the rationality of EFV dosage selection, which may not only maintain efficacy but also reduce the incidence of adverse reactions as well as treatment costs [36–38].
